# Applying Brown and Savulescu: the diachronic condition as excuse

**DOI:** 10.1136/medethics-2019-105684

**Published:** 2019-08-08

**Authors:** Neil Levy

**Affiliations:** 1 Philosophy, Macquarie University, Sydney, New South Wales, Australia; 2 Uehiro Centre for Practical Ethics, University of Oxford, Oxford, UK

**Keywords:** resource allocation

In applied ethics, debates about responsibility have been relentlessly individualistic and synchronic, even as recognition has increased in both philosophy and psychology that agency is distributed across time and individuals. I therefore warmly welcome Brown and Savulescu’s analysis of the conditions under which responsibility can be shared and extended. By carefully delineating how diachronic and dyadic responsibility interact with the long-established control and epistemic conditions, they lay the groundwork needed for identifying how responsibility may be inter-individual and intra-individual.

Unsurprisingly, I don’t agree with every aspect of their rich account (as they anticipate, in discussing my work). I strongly suspect that the privileged place the individual continues to occupy in their taxonomy is a residue of the kind of internalist intuitions which dominate WEIRD (Western, Educated, Industrialised, Rich and Democratic) thinking.^1 2^ However, I won’t pursue this line of thought here (having done so elsewhere^3^). Instead, I apply Brown and Savulescu’s analysis. Moving beyond the programmatic level at which they develop their account, I will show how it provides a basis for excusing many individuals, focusing not on the dyadic (or, as I would prefer, social) level but the diachronic.

Most of the behaviours that can result in ill-health are not risky in one-off instances (as Brown and Savulescu recognise). In fact, many of them are not merely permissible: they may even be a part of a good human life. Perfect self-control on all occasions is not an ideal to aspire to: spontaneity and free-spiritedness are also valuable. The ideal agent will retain control within the sphere of what Kant called the perfect duties (those we are obliged to abide by on every occasion) but it is permissible and perhaps desirable sometimes to drop one’s inhibitions elsewhere. Routine drunkenness is a vice; routine gluttony is (literally) a vice, but overconsumption on a special occasion—or just because one feels like it—is not, not when it fails to form part of a pattern of behaviour.

It is for this kind of reason (in part) that responsibility for ill-health must be diachronic, as Brown and Savulescu agree. However, there is an interesting subset of health-related behaviour that has the following property: though occasional indulgence is fine, the threshold at which such actions form a problematic pattern—that is, a pattern that significantly risks ill-health—is quite low. The clearest example is the use of addictive drugs. The substance abuser who succeeds in abstaining for many weeks or months might undo much of the benefit of abstinence by a single slip, since the slip makes further abstinence extremely difficult (addiction is a chronic relapsing disease, and relapsing months or even years after last use is not uncommon). Similarly, the benefits of abstaining from risky sexual behaviour on most or almost all occasions may be entirely undone by one slip.

Less obviously, the threshold for problematic behaviour is relatively low for overeating. The person who eats sensibly 6 days a week may nevertheless be subject to weight gain and associated problems. Other behaviours are not like this: exercising once a week has benefits even if one is lazy on the other days; still others are problematic in some contexts and not others. In any case, there are a wide class of behaviours with regard to which people may successfully exercise self-control on many occasions but fail to gain any significant benefit from their self-control.

Once we recognise this fact, we can go on to identify individuals who are unlikely to satisfy the diachronic conditions on responsibility. The reason is this: there are particular sets of individuals who face recurrent self-control challenges. For example, poorer people tend to live in obesogenic environments, in which there are many fast food outlets.^4^ This fact entails that locals face many more occasions of temptation for consumption. It is likely that people in such environments typically have reduced control capacities compared to those in wealthier neighbourhoods.^5^ But we don’t need to investigate this factor to conclude that those in poorer environments satisfy the diachronic condition on moral responsibility less well than those in wealthier.

As Brown and Savulescu recognise, capacities for control fluctuate over time. As a consequence, those who face many more temptations can be expected sometimes to encounter them at times when their control capacities are at a low ebb, just by chance. If the threshold for constituting a problematic capacity for consumption is relatively low, an obesogenic environment entails that the threshold is met *and* that it is met in a way that mitigates responsibility: it is met because of facts outside the agents’ control (since they cannot control their environment) and stochastic fluctuations in their capacities for control. What goes for consumption of fast food is true for many other temptations too. For instance, easy availability of addictive drugs is associated with relapse among long-term substance abusers.^6^

Of course, there are potential complications. For instance, might agents in these environments have had what Brown and Savulescu call Golden Opportunities to leave them? For the most part, however, we can ignore these complications: such opportunities are few and far between (as a glance at social mobility statistics can confirm). Thus, attention to the diachronic condition can allow us to assess the typical level of responsibility of large segments of the population for particular health-related outcomes.
